# MicroRNA signatures differentiate Crohn’s disease from ulcerative colitis

**DOI:** 10.1186/s12865-015-0069-0

**Published:** 2015-02-10

**Authors:** Jeremy S Schaefer, Taraq Attumi, Antone R Opekun, Bincy Abraham, Jason Hou, Harold Shelby, David Y Graham, Charles Streckfus, John R Klein

**Affiliations:** Department of Diagnostic and Biomedical Sciences, The University of Texas Health Science Center at Houston School of Dentistry, Houston, TX 77054 USA; Department of Medicine, Baylor College of Medicine, Houston, TX USA; Michael E. DeBakey Veterans Affairs Medical Center, Houston, TX USA; Texas Children’s Hospital, Houston, TX USA; VA HSR&D Center for Innovations in Quality, Effectiveness and Safety, Michael E. DeBakey VA Medical Center, Houston, TX USA

**Keywords:** microRNAs, IBD, Blood, Colon, Saliva

## Abstract

**Background:**

Excessive and inappropriate immune responses are the hallmark of several autoimmune disorders, including the inflammatory bowel diseases (IBD): Crohn’s disease (CD) and ulcerative colitis (UC). A complex etiology involving both environmental and genetic factors influences IBD pathogenesis. The role of microRNAs (miRNAs), noncoding RNAs involved in regulating numerous biological processes, to IBD pathology, in terms of initiation and progression, remains ill-defined. In the present study, we evaluated the relationship between colon, peripheral blood, and saliva whole miRNome expression in IBD patients and non-inflammatory bowel disease (non-IBD) controls to identify miRNAs that could discriminate CD from UC. Quantitative real-time PCR (qRT-PCR) was used to validate and assess miRNA expression.

**Results:**

Microarray analysis demonstrated that upwards of twenty six miRNAs were changed in CD and UC colon biopsies relative to the non-IBD controls. CD was associated with the differential expression of 10 miRNAs while UC was associated with 6 miRNAs in matched colon tissues. CD was associated with altered expression of 6 miRNAs while UC was associated with 9 miRNAs in whole blood. Expression of miR-101 in CD patients and miR-21, miR-31, miR-142-3p, and miR-142-5p in UC patients were altered in saliva.

**Conclusions:**

Our results suggest that there is specific miRNA expression patterns associated with UC versus CD in three separate tissue/body fluids (colon, blood, and saliva). Further, the aberrant miRNA expression profiles indicate that miRNAs may be contributory to IBD pathogenesis, or at least reflect the underlying inflammation. Scrutinizing miRNA expression in saliva and blood samples may be beneficial in monitoring or diagnosing disease in IBD patients. A panel of miRNAs (miR-19a, miR-21, miR-31, miR-101, miR-146a, and miR-375) may be used as markers to identify and discriminate between CD and UC.

**Electronic supplementary material:**

The online version of this article (doi:10.1186/s12865-015-0069-0) contains supplementary material, which is available to authorized users.

## Background

Crohn’s disease (CD) and ulcerative colitis (UC) are the two primary and most prevalent forms of inflammatory bowel disease (IBD). Characterized by inappropriate and exacerbated immune responses within the gastrointestinal tract, the nature and location of the inflammatory changes defines each respective disease. Despite years of research, the exact cause of IBD is still relatively poorly understood. Nevertheless, it is clear that many factors, including genetic and epigenetic predispositions, the gut microbiota content, and environmental injuries or exposures, contribute significantly to the disease process. Linkage analysis and genome wide association studies (GWAS) have uncovered over 100 loci that have significant association with IBD [1-3]. Cytokines (IL-17), cytokine receptors (IL-23 receptor), and bacterial response elements (CARD15/NOD2, ATG16L1) are just a few of the pathways that have been found to be either mutated or otherwise altered in IBD patients and mouse models of chronic intestinal inflammation [2-5].

MicroRNAs (miRNAs), single stranded RNA molecules of 19–25 nucleotides, are poised to make significant contributions to defining the multifactorial etiology and pathobiology of IBD. Initially discovered in the early 1990s, this novel class of noncoding RNAs regulates gene expression post-transcriptionally to repress translation and/or promote mRNA degradation [6-9]. The biological footprint of miRNAs is widespread; over 30% of the genome is predicted to be actively regulated by miRNAs and studies have shown that miRNAs are involved in the control of a variety of normal cellular events including differentiation, organogenesis, and metabolism [10,11]. Further, aberrant expression of miRNAs has been associated with a growing number of disease states, including cancer and autoimmune diseases [12-17].

However, the consequence of how the alterations in miRNA expression occur and contribute to disease pathobiology remains mostly intangible at the moment. Understanding the role of miRNAs in the regulation of inflammation is an area of importance that may have broad significance to understanding the pathogenesis of IBD as well as a number of other diseases. Several studies have identified miRNAs that are associated with IBD in intestinal tissues and peripheral blood specimens [18-24]. In a previous study involving the interleukin-10 knockout (IL-10−/−) mice, a mouse model of chronic intestinal inflammation, we demonstrated selective dysregulation of miRNAs in colon tissue and peripheral blood leukocytes [25]. We therefore postulated that miRNA expression would be similarly disrupted in IBD patients with the possibility that some of the same miRNAs might have altered expression. The purpose of this study was to identify differentially expressed miRNAs that could selectively discriminate CD from UC and healthy controls using colon, blood, and saliva specimens. A further goal was to determine how the tissue microenvironment affected miRNA expression.

## Results

### Whole miRNome expression analysis in human IBD colon biopsies

**Figure 1:**
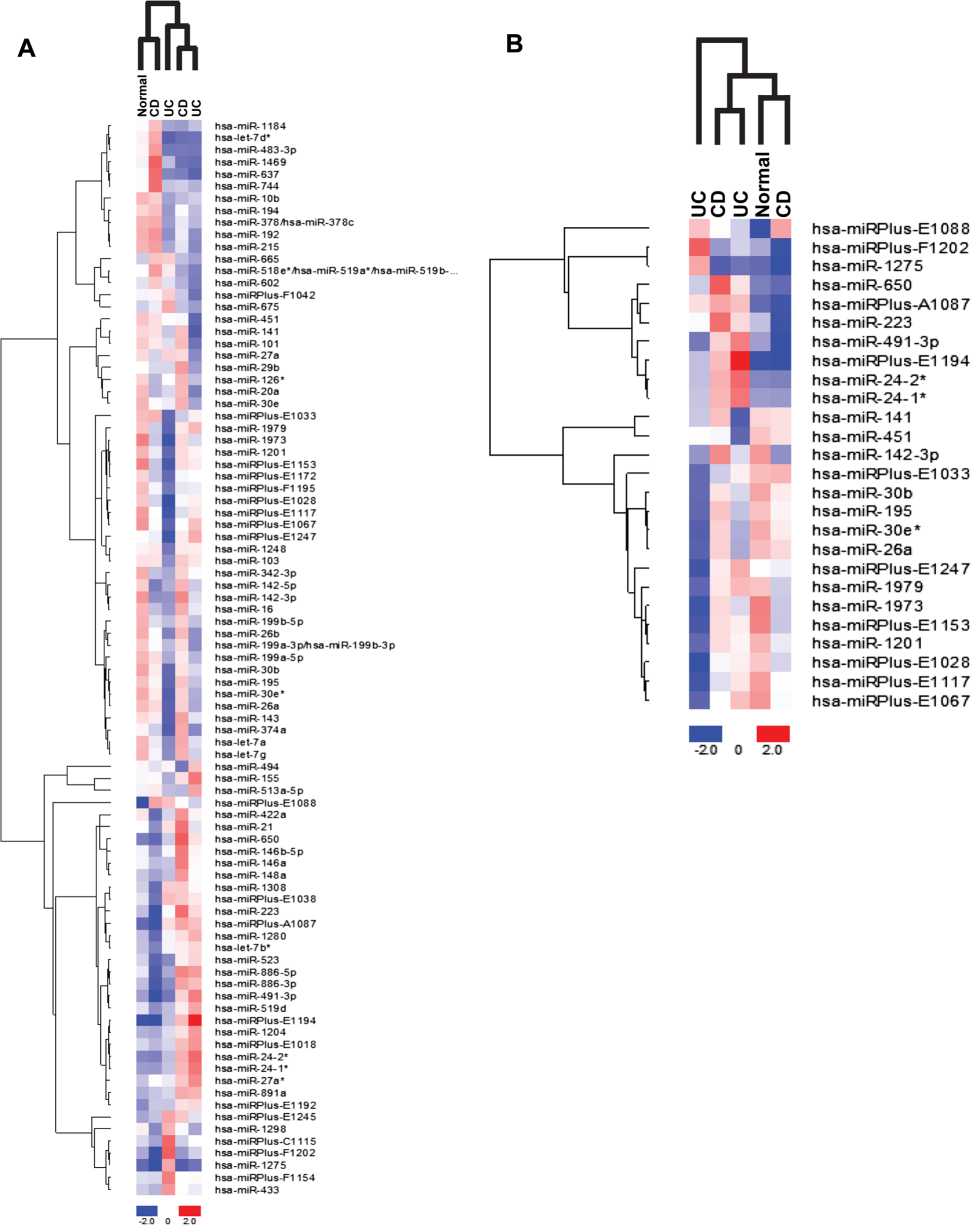
**Heat map and unsupervised hierarchical clustering of miRNAs in IBD colon biopsies indicate dysregulation of miRNA expression.** The miRCURY LNA microarray microRNA profiling service was used to examine miRNA expression in colon biopsies from Normal, CD, and UC subjects. **(A)** Heat map of fold changes > 0.5. **(B)** Heat map of fold changes > 3.0. Red color represents an expression level above mean, blue color represents expression lower than the mean. A Delta Log Median ratio of +/−1.0 is equal to a fold change of +/− 2.0.

To systematically analyze miRNA expression in IBD patients and identify candidate miRNAs for subsequent validation, expression analysis using oligonucleotide microarrays was initially performed on a small number of colon tissue biopsies from CD, UC, and non-IBD (Normal, NL) patients. Among the over 600 miRNA probes, the microarray detected 89 miRNAs with fold changes > 0.5 (Figure 1A) and 26 miRNAs with fold changes > 3.0 over the mean (Figure 1B).

### miRNAs are differentially expressed in CD and UC intestinal biopsies

From the initial microarray screen, we selected 12 miRNAs for further validation. Among the qualities that factored into the inclusion of select miRNAs for validation was overall expression pattern, magnitude of expression, and previous associations with inflammation or disease. In terms of overall expression, we looked to include miRNAs that had consistent elevated or reduced expression in either the two CD or two UC biopsies relative to the non-IBD biopsy. miR-21, miR-26a, miR-101, miR-142-3p, miR-142-5p, miR-146a, miR-155, miR-223, and miR-494 were identified as candidate miRNAs in this manner. As an example miR-142-5p expression was decreased in both UC biopsies relative to the non-IBD biopsy. miR-26, miR-142-3p, and miR-223 were chosen as they were among the few miRNAs that had fold changes > 3.0 over the mean (Figure 1B). miR-19a, miR-31, and miR-375 were included as these miRNAs were elevated in our previous study with IL-10−/− mice or are associated with disease [25].

**Figure 2:**
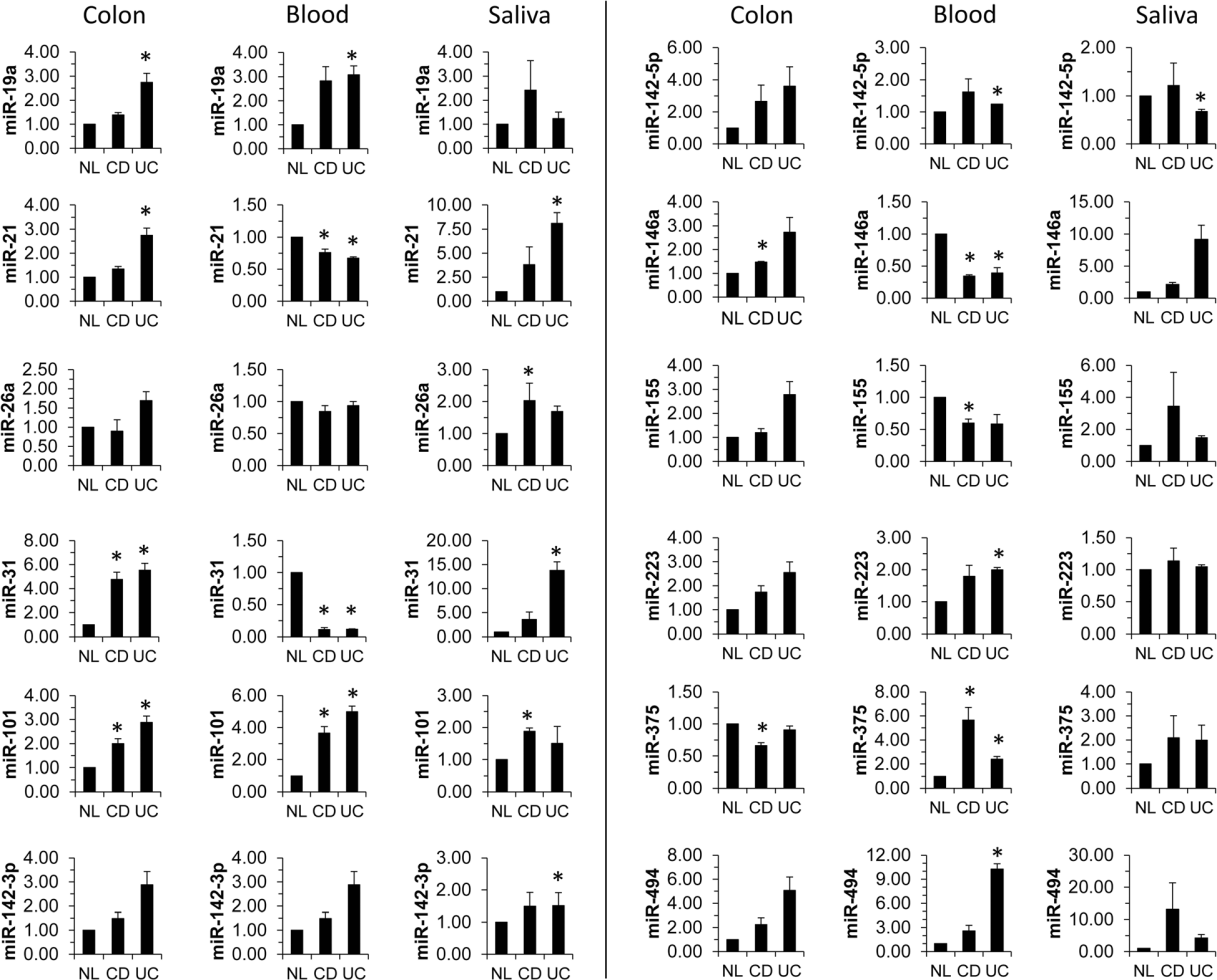
**miRNA expression is altered in colon, blood, and saliva from Crohn’s disease and ulcerative colitis subjects.** Total RNA from NL, CD, and UC colon biopsies was pooled and used for TaqMan qRT-PCR analysis for the indicated miRNAs. miRNA expression was normalized to U6 expression. Statistical significance was calculated using the Student’s *t*-test relative to the healthy NL control. At least 23 distinct specimens were included in each pooled group of colon biopsies. 30 and 5 distinct specimens were included in each pooled group of blood and saliva samples, respectively.

To validate miRNA expression, three separate condition-specific groups (NL, CD, or UC ) were each made by pooling total RNA from the corresponding NL, CD, or UC patient groups (n ≥ 20 biopsies per group) and analyzed via qRT-PCR for the selected miRNAs. In these pooled colon biopsies miR-31, miR-101, and miR-146a were significantly elevated in the CD samples while miR-375 was significantly reduced relative to the NL samples (Figure 2, column 1 & 4; *p* < 0.05). miR-19a, miR-21, miR-31, and miR-101 levels were significantly elevated in UC colon biopsies relative to the NL samples (Figure 2, column 1 & 4; *p* < 0.05). Secondary analysis using a Bonferroni correction (α = 0.05; n = 12) revealed that miR-146a was statistically elevated in CD biopsies (*p* < 0.004).

### Circulating miRNAs are differentially expressed in CD and UC blood

To advance our knowledge in using less-invasive methods to screen for IBD, we characterized the extra-intestinal expression of miRNAs in peripheral blood. Peripheral blood is frequently used as a diagnostic tool for a varied number of diseases and conditions from simple cholesterol screenings to advanced genetic screens. Currently, there is not a single blood test that can make a diagnosis of IBD on its own merits; rather, multiple tests are required to come to a diagnosis of IBD. Thus, we explored the possibility of using whole blood miRNA expression profiling as an IBD diagnostic tool.

In order to determine if the miRNA profile uncovered in the colon biopsies could be replicated in a circulating extra-intestinal body fluid, whole blood was collected to examine miRNA expression levels. As above, total RNA from the blood specimens were pooled into three separate condition-specific groups (NL, CD, or UC) and analyzed for miRNA expression via qRT-PCR. A total of ten miRNAs (6 with CD and 9 with UC) had statistically significant altered expression in IBD peripheral blood pools versus non-IBD blood pools. In CD blood samples, miR-21, miR-31, miR-146a, and miR-155 were significantly reduced while miR-101 and miR-375 were significantly elevated relative to the NL samples (Figure 2, column 2 & 5; *p* < 0.05). miR-21, miR-31, and miR-146a were statistically significantly reduced and miR-19a, miR-101, miR142-5p, miR-223, miR-375, and miR-494 were statistically significantly elevated in UC blood samples relative to the NL samples (Figure 2, column 2 & 5; *p* < 0.05). Of these, miR-19a, miR-31, miR-101, miR-142-5p, miR-146a, miR-155, miR-223, miR-375, and miR-494 had not previously been associated with IBD to our knowledge. However, miR-21 has previously been shown to be differentially expressed in IBD [26,27]. In our previous study with IL-10−/− mice, miR-19a, miR-31, miR-101, miR-142-5p, miR-146a, miR-155, miR-223, and miR-375 were selectively dysregulated in whole blood of mice with mild intestinal pathology [25]. Secondary analysis using a Bonferroni correction (α = 0.05; n = 12) revealed that miR-21, miR-31, miR-142-5p, and miR-146a expression was significantly altered in CD and UC blood specimens (*p* < 0.004).

### Evaluation of miRNA expression in oral fluid

Saliva is an important body fluid containing enzymatic proteins to begin digestion and antimicrobial proteins for immune protection. Similar to the peripheral blood, during pathophysiological states, such as cancer and inflammation, the composition of saliva can become altered as a reflection of these conditions [28,29]. Moreover, saliva provides a more accurate ‘real-time read-out’ than serum to screen and monitor the health status of patients. In addition, evidence suggests that saliva contains a transcriptome of thousands of mRNAs including miRNA [30-34]. Furthermore, due to the non-invasive nature of saliva collection for analytical purposes, saliva collection does not compromise a patient’s skin barrier nor does it require sedation, saliva is the ideal fluid for procuring multiple isolates to monitor disease progression. In our study, 5 miRNAs (1 CD and 4 UC) had statistically significant altered expression in pooled saliva samples from IBD patients relative to the non-IBD controls. In pooled saliva samples, miR-101 was significantly elevated in CD relative to the NL samples while miR-21, miR-31, and miR-142-3p were significantly elevated in UC relative to the NL samples (Figure 2, column 3; *p* < 0.05). miR-142-5p expression was significantly lowered in UC saliva samples relative to the NL samples (Figure 2, column 6; *p* < 0.05). No saliva specimens were statistically significant following a secondary analysis using a Bonferroni correction (α = 0.05; n = 12; *p* < 0.004).

To our knowledge this represents the first report of salivary miRNA diagnostics in IBD patients. Thus, examining the expression of miR-101, miR-21, miR-31, miR-142-3p, and miR-142-5p in saliva may be able to assist in diagnosing IBD.

The pooled colon, pooled blood, and pooled saliva specimen results are summarized in Additional file 1: Table S1 and Table S2 for CD and UC, respectively. miRNA expression profiles were altered in the extra-intestinal body fluids suggesting that these sources may effectively reflect the intestinal inflammation in IBD. miR-101, in particular, may be a key miRNA regulator in IBD, as it was statistically elevated in all three tissues from CD patients and two of the three UC tissues relative to the NL tissues.

### miRNA expression is differentially expressed in matched colon biopsies from CD and UC subjects

**Figure 3:**
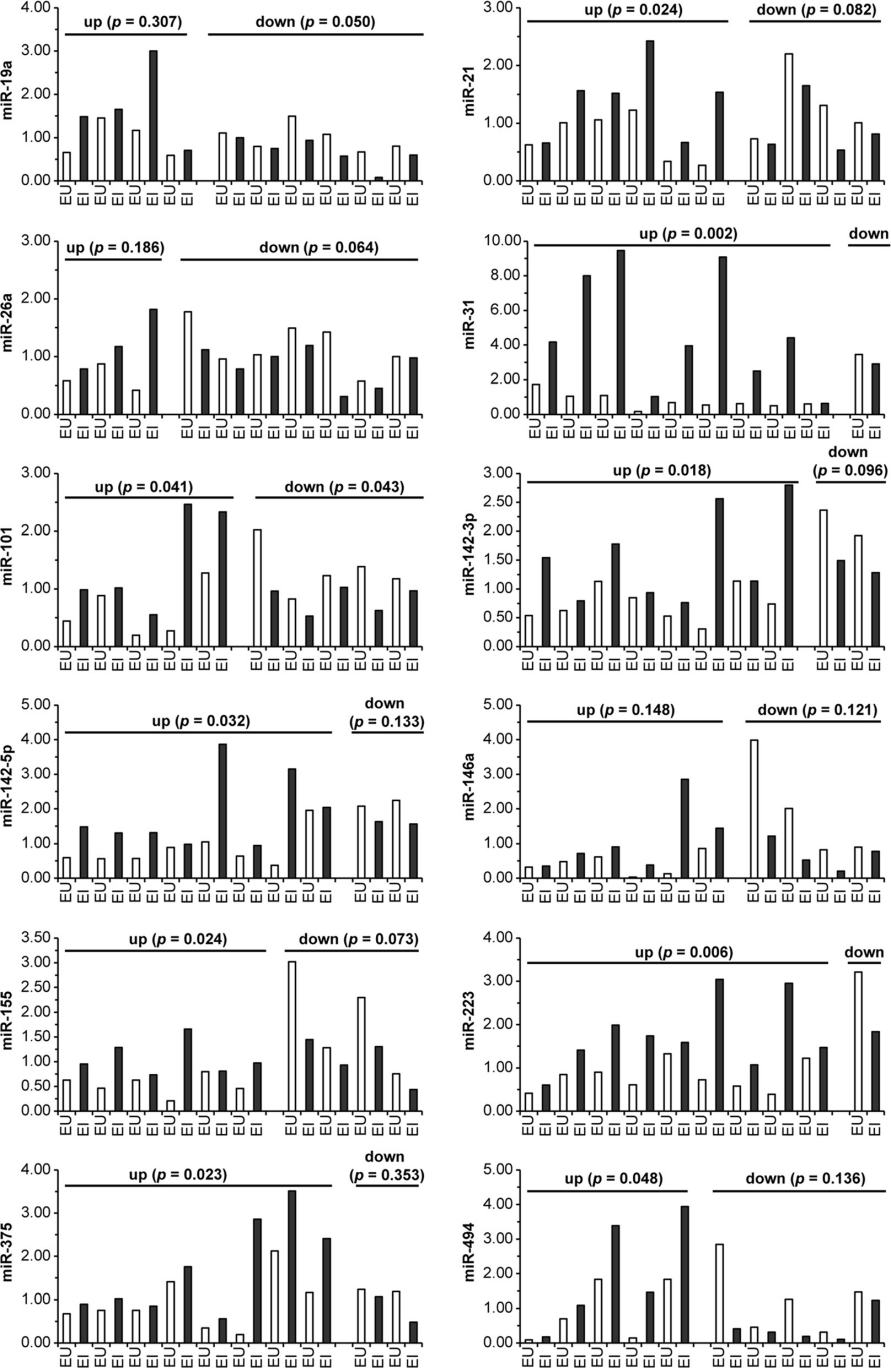
**miRNA expression is differentially expressed in matched colon biopsies from Crohn’s disease subjects.** Total RNA was isolated from matched endoscopically uninvolved (EU) and endoscopically involved (EI) colon biopsies from CD subjects. The RNA samples were used for TaqMan qRT-PCR analysis for the indicated miRNAs. miRNA expression was normalized to U6 expression. Pairings were subdivided according to miRNA expression trends. Statistical significance was calculated using the Student’s paired *t*-test relative to the endoscopically uninvolved CD tissue.

**Figure 4:**
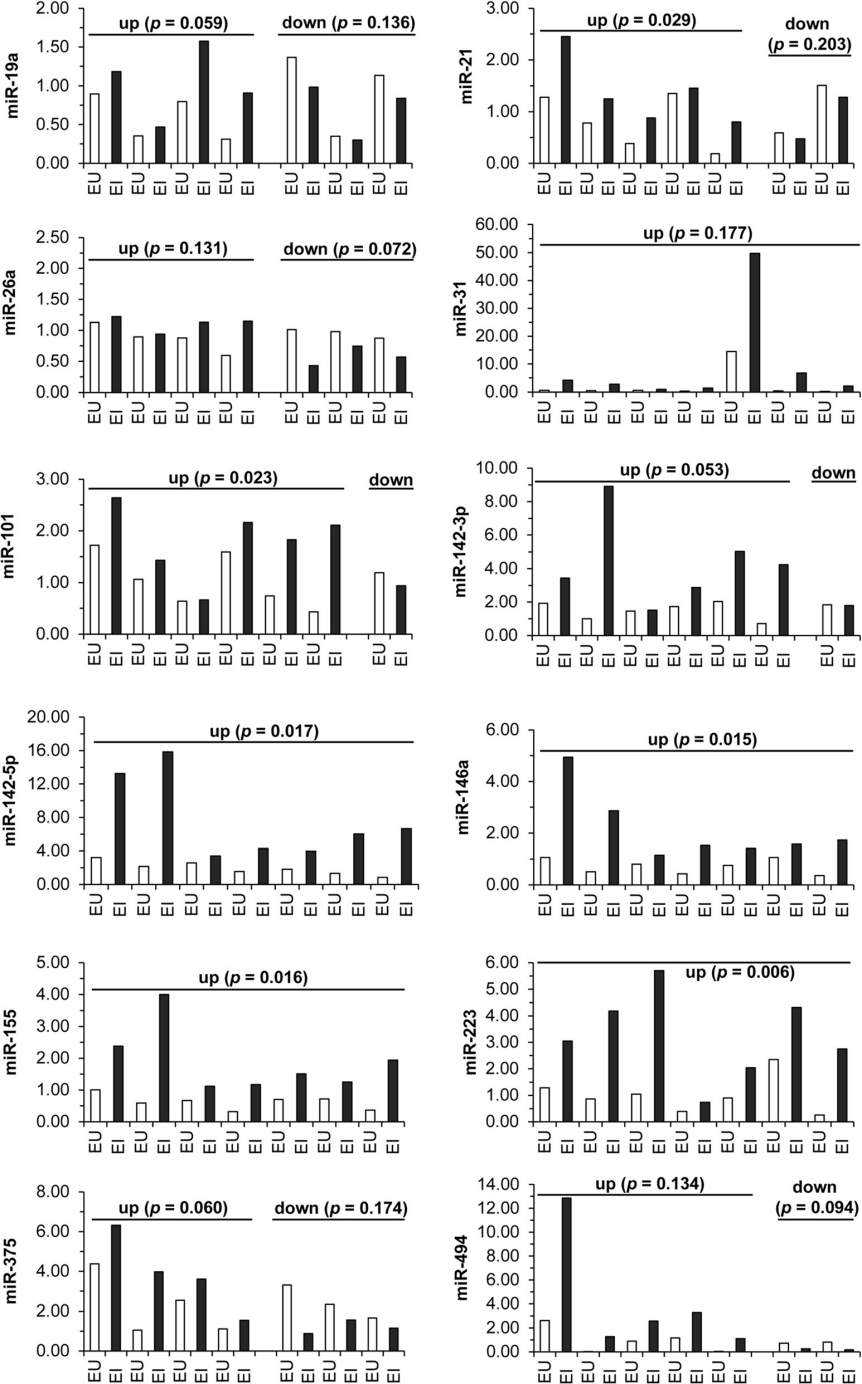
**miRNA expression is differentially expressed in matched colon biopsies from ulcerative colitis subjects.** Total RNA was isolated from matched endoscopically uninvolved (EU) and endoscopically involved (EI) colon biopsies from UC subjects. The RNA samples were used for TaqMan qRT-PCR analysis for the indicated miRNAs. miRNA expression was normalized to U6 expression. Pairings were subdivided according to miRNA expression trends. Statistical significance was calculated using the Student’s paired *t*-test relative to the endoscopically uninvolved UC tissue.

As CD in particular is characterized by patchy areas of involved, inflamed tissues, we next sought to determine if a spatial relationship exists in miRNA expression patterns between these pockets of intestinal pathology and non-involved tissue in CD and UC. Paired endoscopically uninvolved (EU) and endoscopically involved (EI) colon biopsies from the same CD (Figure 3) or UC patients (Figure 4) were drawn from the previously analyzed pooled groups to examine their miRNA profiles. In the CD paired colon biopsies, nine miRNAs (miR-21, miR-31, miR-101, miR-142-3p, miR-142-5p, miR-155, miR-223, miR-375, and miR-494) were elevated at a statistically significant level in the endoscopically involved colonic tissue in comparison to the endoscopically uninvolved colonic tissue (Figure 3, Table 1). miR-21, miR-101, miR-142-5p, miR-146a, miR-155, and miR-223 were elevated at a statistically significant level in the UC endoscopically involved colonic tissue in comparison to the endoscopically uninvolved colonic tissue (Figure 4, Table 1). Although miR-31 was the most highly expressed miRNA in both the CD and UC matched pairs, it was statistically significant in only the CD pairs.

**Table 1:** **Summary of microRNA changes in paired colon biopsies from Crohn’s disease and ulcerative colitis subjects**

	**Crohn’s disease**			**Ulcerative colitis**		
**miRNA**	**N (Pairs)**	**Reduced (** ***p*** **-value)**	**Elevated (** ***p*** **-value)**	**N (Pairs)**	**Reduced (** ***p*** **-value)**	**Elevated (** ***p*** **-value)**
**miR-19a**	10	6 (0.050)*	4 (0.307)	7	3 (0.136)	4 (0.059)
**miR-21**	10	4 (0.082)	6 (0.024)*	7	2 (0.203)	5 (0.029)*
**miR-26a**	10	7 (0.064)	3 (0.186)	7	3 (0.072)	4 (0.131)
**miR-31**	10	1 (ND)	9 (0.002)*	7	0 (ND)	7 (0.177)
**miR-101**	10	5 (0.043)	5 (0.041)*	7	1 (ND)	6 (0.023)*
**miR-142-3p**	10	2 (0.096)	8 (0.018)*	7	1 (ND)	6 (0.053)
**miR-142-5p**	10	2 (0.133)	8 (0.032)*	7	0 (ND)	7 (0.017)*
**miR-146a**	10	4 ( 0.121)	6 (0.148)	7	0 (ND)	7 (0.015)*
**miR-155**	10	4 (0.073)	6 (0.024)*	7	0 (ND)	7 (0.016)*
**miR-223**	10	1 (ND)	9 (0.006)*	7	0 (ND)	7 (0.006)*
**miR-375**	10	2 (0.353)	8 (0.023)*	7	3 (0.174)	4 (0.060)
**miR-494**	10	5 (0.136)	5 (0.048)*	7	2 (0.094)	5 (0.134)

ND, not determined due to insufficient number of samples in group.

*statistically significant values, *p* < 0.05.

**Table 2:** **Characteristics of IBD patients and controls**

			**CD**	**UC**	**Controls**
No. of patients (total)			42	41	35
Patient specimens					
	Pinch biopsies		33	23	34
	Blood		30	30	30
	Saliva		5	5	5
Sex					
	Male, n (%)		20 (47.6)	28 (68.3)	13 (37.1)
	Female, n (%)		22 (52.4)	13 (31.7)	22 (62.9)
Age (y)					
	Mean		42.7	47.6	56.6
		Male	46.7	50.3	55.4
		Female	39	41.8	57.4
	Range		18-71	18-88	26-82
		Male	18-71	27-88	26-82
		Female	19-65	18-62	34-75

In the miRNA microarray analysis, several of the non-annotated, human miRPlus miRNA candidates had altered expression (Figure 1A-B). miRPlus miRNA candidates are predicted miRNA sequences derived from Exiqon’s database of proprietary material, database mining, and publications. We investigated the expression of the top seven of these miRPlus miRNAs that were altered in the microarray analysis. In the matched CD biopsies, miRPlus-E1067 and miRPlus-E1117 were statistically significantly reduced while miRPlus-E1028 and miRPlus-F1202 were statistically significantly elevated in the endoscopically involved colonic tissue in comparison to the endoscopically uninvolved colonic tissue (Additional file 2: Figure S1). In the matched UC biopsies, miRPlus-E1067, miRPlus-E1088, and miRPlus-E1117 were statistically significantly reduced in the endoscopically involved colonic tissue in comparison to the endoscopically uninvolved colonic tissue (Additional file 3: Figure S2).

To confirm the reproducibility of the qRT-PCR analysis, biopsies were run in replicate; results for individual runs for 9 of the 12 miRNAs analyzed are shown (Additional file 4: Figure S3A). This confirms that there is little intra-sample variation between qRT-PCR runs.

We next decided to examine the expression of three miRNAs (miR-31, miR-146a, and miR-375) in pooled endoscopically uninvolved versus endoscopically involved IBD patient biopsies to determine if this panel of miRNAs could be used to separate CD from UC. A general pattern (Table 1) emerged for these 3 miRNAs in the paired analysis; miR-31 and miR-375 expression was increased at a statistically significant level in CD but not UC, while miR-146a is increased at a statistically significant level in UC but not CD. The CD and UC pairs from Figures 3 and 4 were pooled into endoscopically uninvolved versus endoscopically involved groups of CD or UC, respectively (CD EI, CD EU, UC EU, UC EI). Elevated expression of miR-31, miR-146a, and miR-375 was present in the CD pool while miR-31 and miR-146a were elevated in UC (Additional file 4: Figure S3B).

### Roquin-1 and ATG16L1 expression is reduced in matched colon biopsies from CD but not UC subjects

To better understand the relationship that exists between miRNA expression and potential gene targets of interest, we used web-based target prediction programs (miRDB and TargetScan) to identify potential miRNA target genes (Additional file 1: Table S3). Of the predicted potential miRNA target genes, Roquin-1 (*RC3H1*), a RING finger E3 ligase with immunoregulatory properties in mice, was a common target for seven of the miRNAs with elevated expression. Autophagy related 16-like 1 (*ATG16L1*), one of the most commonly detected genetic variations in CD patients, was predicted to be a target of miR-142-3p [35,36]. Our unpublished observations and others have experimentally confirmed ATG16L1 as a regulatory target of miR-142-3p and miR-93-5p [37,38].

**Figure 5:**
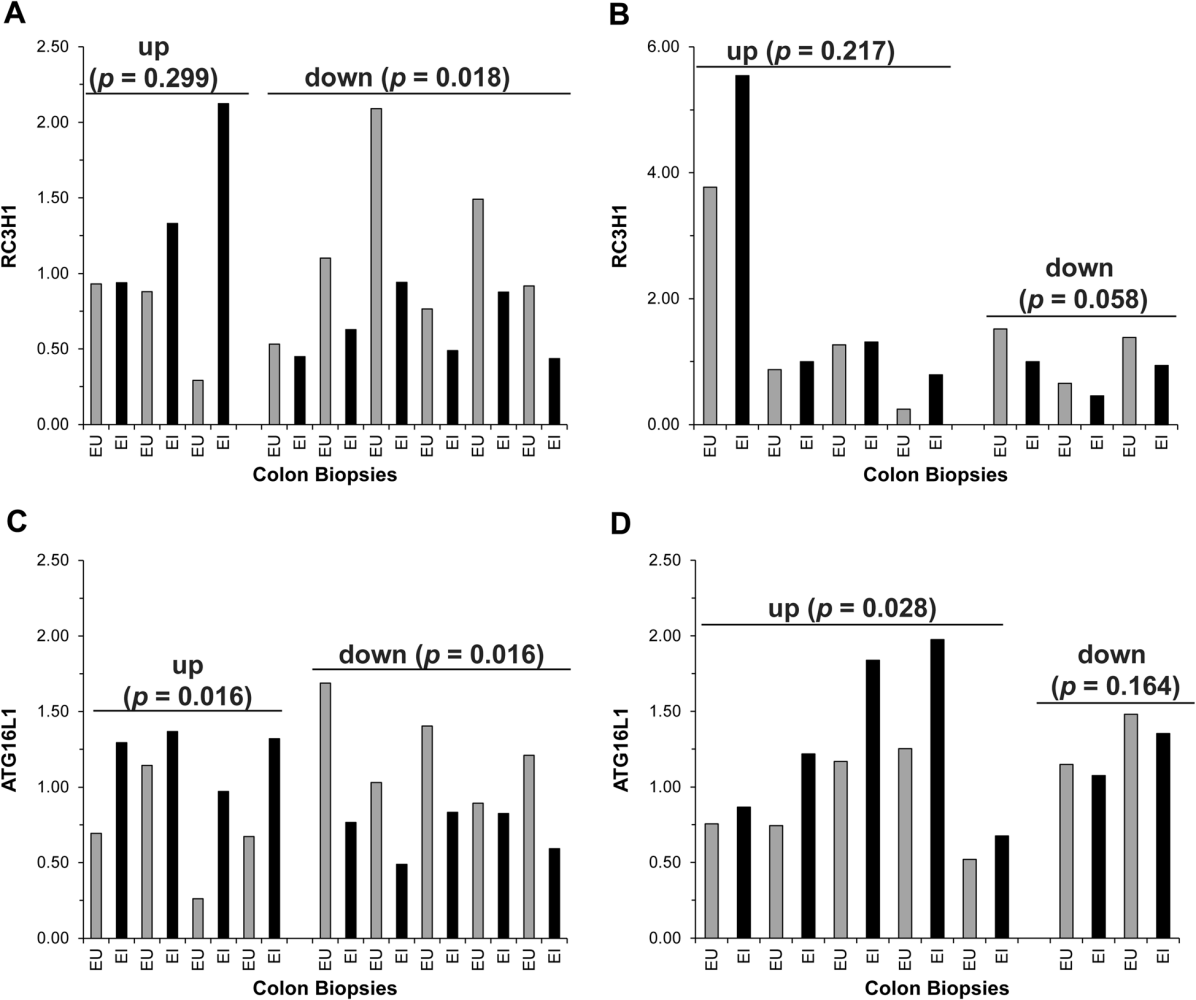
**Roquin-1 and ATG16L1 expression is differentially expressed in a subset of CD and UC subjects.** Total RNA from matched endoscopically uninvolved (EU) and endoscopically involved (EI) colon biopsies from **(A, C)** CD and **(B, D)** UC subjects was used to analyze Roquin-1 or ATG16L1 expression via qRT-PCR. Gene expression was normalized to GAPDH. Pairings were subdivided according to Roquin-1 or ATG16L1 expression trends. Statistical significance was calculated using the Student’s paired *t*-test relative to the endoscopically uninvolved IBD tissue.

Analysis of Roquin-1 mRNA expression in the paired endoscopically uninvolved (EU) versus the endoscopically involved (EI) CD colon biopsies revealed statistically significant decreased expression of Roquin-1 in a subset of CD patients (Figure 5A). There was no appreciable difference in UC (Figure 5B). ATG16L1 expression was statistically decreased in a subset of CD patients (Figure 5C). Conversely, ATG16L1 expression was statistically increased in a subset of UC (Figure 5D).

## Discussion

In the present study into the miRNA regulatory network of IBD, we were able to identify miRNAs (miR-19a, miR-21, miR-31, miR-101, miR-146a, and miR-375) that had statistically significant altered expression in pooled colon biopsy samples with several other miRNAs just outside the significance threshold (miR-26a, miR-142-3p, miR-155, miR-223, and miR-494; *p* ≤ 0.1). Of these miRNAs, miR-21, miR-31, miR-146a, and miR-375 have been identified in previous studies examining miRNA expression in CD and UC colon biopsies [18-21]. Further, these miRNAs are implicated in inflammation and cancer with miR-21 and miR-375 functioning as oncomiRs while miR-146a has been reported to be a tumor suppressor [39-41]. miR-31 is more complicated in that it has been reported to be both an oncomiR in lung cancer, and a tumor suppressor in breast cancer [42,43]. The increased expression of miR-146a and possibly miR-31 seem to be attempts to rein in the chronic inflammatory response in IBD. Previous studies that established miR-146a functionally opposing the effects of miR-155 via regulation of inflammatory signaling proteins, particularly NF-κB, support this [44-47]. miR-155 overexpression in mice results in a myeloproliferative disorder [44]. Conversely, miR-146a knockout mice develop hyperinflammatory and immunoproliferative disorders [45-47]. This suggests that, in IBD, the balance between these miRNAs with seemingly opposing functions is tilted and begs the question as to why the pro-inflammatory miRNAs win.

We additionally identified two miRNAs, miR-19a and miR-101, that have not previously been associated with IBD. miR-19a is a member of the miR-17-92 cluster and has been shown to be overexpressed in T-cell acute lymphoblastic leukemia and multiple myeloma where it was revealed that miR-19a negatively regulates the expression of CYLD and SOCS-1 respectively to promote cell survival and pathogenesis [48,49]. miR-101 has been associated with inflammation as a negative regulator of inducible costimulator (ICOS) and cancer cell stemness as a negative regulator of the corepressor C-terminal binding protein-2 (CtBP2) [50,51]. Once again, two miRNAs with seemingly opposing targets, miR-19a is pro-inflammatory while miR-101 is anti-proliferative, are both elevated in IBD.

Further, we were able to demonstrate that CD was associated with the differential expression of 10 miRNAs in a statistically significant manner (9 elevated, 1 decreased) while 6 miRNAs were elevated in a statistically significant manner in UC when comparing matched endoscopically uninvolved colon biopsies with endoscopically involved colon biopsies. Of these miRNAs, miR-21, miR-101, miR-142-5p, miR-155, and miR-223 had overlapping expression patterns between CD and UC. miR-146a was elevated in a statistically significant manner only in UC in the paired analysis while miR-19a, miR-31, miR-142-3p, miR-375, and miR-494 were altered in a statistically significant manner in CD.

Taken together, our analysis of the pooled colon samples versus the matched colon biopsies provides crucial but distinct information regarding the role of miRNAs in IBD. The former analysis suggests that a panel of miRNAs (miR-19a, miR-21, miR-31, miR-101, miR-146a, and miR-375) may be used as markers to identify and discriminate between CD and UC. The latter analysis provides more mechanistic information regarding miRNA involvement in disease pathology. Namely, it points to the underlying pathways that the miRNAs are altering to cause or promote disease. As an example, the overexpression of miR-31 and miR-146a, both of which target CD40L (predicted or experimentally confirmed), in the matched samples suggests that the CD40:CD40L costimulatory pathway plays an important role in promoting localized inflammation; CD40 and CD40L are elevated in IBD [52]. With further studies, we may even be able to distinguish patients at higher risk of colon cancer based on miRNA analysis.

As a proof of concept, we analyzed miRNA expression in the saliva of IBD patients. This new frontier of RNA diagnostics in oral fluids is being pioneered in the detection of cancer [33,53-56]. Although salivary miRNAs are emerging as a novel class of biomarkers in cancer, they have yet to be examined in other non-cancer diseases. In our study, we were able to detect differences in miRNA expression in the oral fluid from IBD patients. miR-21 in particular is a ubiquitous miRNA associated with many diseases and is an oncomiR [57,58]. Likewise, miR-31 and miR-101 have been implicated in cancer as well with the former being described as an oncomiR while the latter behaves as a tumor suppressor [43,59,60]. Finally, our results identified a potentially new IBD associated gene. In a subset of CD patients, *RC3H1* expression was reduced in statistically significant manner in a subset of the endoscopically involved versus endoscopically uninvolved matched colony biopsy pairs. In mouse models deficient for *Rc3h1*, either the sanroque missense mutation (*Rc3h1*^*san/san*^) mouse or a genetrap knockout (*Rc3h1*^*gt/gt*^) mouse, an acute small intestinal inflammation develops among other phenotypes [50,61,62]. Roquin-1 is involved in the post-transcriptional regulation of mRNA. Roquin-1 localizes to P bodies and stress granules and plays a role in regulating mRNA turnover, and has been shown to regulate the expression of T cell coactivators, specifically ICOS and OX40 as well as the cytokines TNFα and IL-17. Given this role and the number of altered IBD-related miRNAs predicted or confirmed to target Roquin-1 (Additional file 1: Table S3), the importance of Roquin-1 in intestinal inflammation and human IBD necessitates further study.

This study also affirms the use of the IL-10−/− mouse as an effective model of IBD as miRNAs identified as differentially expressed in the CD and UC human samples were shared with the IL-10−/− mice [25]. Although there are frequently difficulties in going from bench to bedside, at least in this instance it seems that the IL-10−/− mouse model faithfully recreates some aspects of IBD.

## Conclusions

In summary, selective sets of miRNA expression profiles may serve to separate CD and UC diagnostically. In this study, differentially expressed miRNAs in endoscopically involved versus endoscopically uninvolved CD and UC colon biopsies were discovered that indicate that a distinct miRNA profile exists within the intestinal microenvironment of IBD patients. Further, these study results provide support for the feasibility of using saliva and blood as diagnostic tools and advances the search for viable early detection methods for IBD. Importantly, this is the first report of altered miRNA expression in saliva samples from CD and UC patients.

## Methods

### Patients and controls

Fresh-frozen colonic mucosa was sampled via endoscopic pinch biopsies from the following groups: 1) CD, 2) UC, and 3) normal, healthy individuals scheduled for clinically indicated colonoscopies unrelated to IBD. For the CD and UC patients, one or two pinch biopsies were taken from endoscopically uninvolved appearing mucosa (EU) and a similar number from endoscopically involved (EI) tissue near the other site when possible. The pinch biopsies were immediately placed in RNALater for subsequent RNA isolation and analysis of miRNA expression. Blood and saliva samples were collected from the same groupings above albeit not the same individuals as from which the pinch biopsies were collected. Whole blood was collected in PAXgene Blood RNA Tubes (PreAnalytiX, Switzerland) while unstimulated saliva was collected in vessels containing the RNAprotect Saliva reagent (Qiagen, Valencia, CA). In total, 195 patient samples have been assessed from 35 controls, 42 CD, and 41 UC patients and broken down as: 23 UC pinch biopsies (7 matched pairs), 33 CD pinch biopsies (10 matched pairs), 34 normal pinch biopsies; 30 blood samples per group; and 5 saliva samples per group. All patient samples were collected under the auspices of protocols approved by The University of Texas Health Science Center at Houston Committee for the Protection of Human Subjects (the Institutional Review Board at UTHealth) and the Institutional Review Board for Baylor College of Medicine and Affiliated Hospitals. Written informed consent and informed assent (to level of age-related understanding) was obtained after the attending physician introduced the opportunity for study participation to the participants, or where appropriate, to the legal representative/parent(s) of the participants. Patient characteristics are detailed in Table 2.

### Isolation of RNA and real-time quantitative PCR (qRT-PCR)

Total RNA was isolated from colon biopsies using the miRNeasy Minikit (Qiagen) according to the manufacturer’s instructions. The RNeasy Protect Saliva Minikit (Qiagen) was used to isolate total RNA from the saliva samples with a minor modification recommended by the manufacturer. The PAXgene Blood miRNA Kit (PreAnalytiX) was used to isolate total RNA from the blood samples. cDNA was synthesized using either the High-Capacity cDNA Reverse Transcription Kit or the Taqman microRNA Reverse Transcription Kit (Applied Biosystems, Grand Island, NY). miRNA expression was quantified using TaqMan microRNA assays with the TaqMan Universal Master Mix II, No UNG reagent. To measure Roquin-1 and GAPDH transcript levels, either the Power SYBR Green PCR Master Mix (Applied Biosystems) or the TaqMan Universal Master Mix II, No UNG (Applied Biosystems) was used according to the manufacturer’s instructions. Samples were analyzed using the StepOnePlus real-time thermal cycler (Applied Biosystems) and software. Relative gene expression was calculated using the 2-^ΔΔCt^ method and normalized to either GAPDH or U6 snRNA [63]. Roquin-1 and GAPDH gene specific primers were designed and purchased from Integrated DNA Technologies (Coralville, IA): Roquin-1 Forward, 5′-ACCAACCTTGCCTCCTACCT-3′, Roquin-1 Reverse, 5′-TAATCGCTGGTCCCTCATTC -3′; GAPDH Forward, 5′-TGCACCACCAACTGCTTAGC-3′, GAPDH Reverse, 5′-GGCATGGACTGTGGTCATGAG-3′. Taqman assays for mature miRNAs were purchased from Applied Biosystems.

### miRNA Microarray

Total RNA (~1,000 ng each) from 1 healthy normal, 2 CD, and 2 UC colon biopsies were submitted to Exiqon (Woburn, MA) for miRNA expression analysis via the miRCURY LNA microarray microRNA profiling service. The miRNA array data has been deposited in the NCBI Gene Expression Omnibus (GEO) under the entry series GSE53867.

### Statistical analysis

The statistical significance of data from these studies was determined using either an unpaired, two-sided Student’s t test (pooled samples) or a paired, two-sided Student’s t test (matched samples) in Excel. Data are presented as mean + SEM. Differences were considered statistically significant if p ≤ 0.05. A secondary analysis using the Bonferroni correction was included; differences were considered statistically significant if p ≤ 0.004.

## Additional files

Additional file 1: Table S1. MicroRNAs with statistically significant altered expression in Crohn’s disease by tissue site from pooled samples. **Table S2**. MicroRNAs with statistically significant altered expression in ulcerative colitis by tissue site from pooled samples. **Table S3**. Potential Targets for miRNAs with Elevated Expression in IBD. Additional file 2: Figure S1. miRPlus miRNA expression is altered in matched colon biopsies from Crohn’s disease subjects. Total RNA was isolated from matched endoscopically uninvolved (EU) and endoscopically involved (EI) colon biopsies from CD subjects. The RNA samples were used for SYBR Green qRT-PCR analysis for the indicated miRNAs. miRNA expression was normalized to U6 expression. Pairings were subdivided according to miRNA expression trends. Statistical significance was calculated using the Student’s paired *t*-test relative to the endoscopically uninvolved CD tissue. Additional file 3: Figure S2. miRPlus miRNA expression is altered in matched colon biopsies from ulcerative colitis subjects. Total RNA was isolated from matched endoscopically uninvolved (EU) and endoscopically involved (EI) colon biopsies from UC subjects. The RNA samples were used for SYBR Green qRT-PCR analysis for the indicated miRNAs. miRNA expression was normalized to U6 expression. Pairings were subdivided according to miRNA expression trends. Statistical significance was calculated using the Student’s paired *t*-test relative to the endoscopically uninvolved UC tissue. Additional file 4: Figure S3. Specificity and reproducibility of miRNA expression colon biopsies. (A) Replicate runs of RNA samples from matched endoscopically uninvolved versus endoscopically involved CD patient biopsies. Samples were analyzed via TaqMan qRT-PCR for the indicated miRNAs. (B) qRT-PCR analysis of miR-31, miR-146a, and miR-375 in pooled endoscopically uninvolved versus endoscopically involved CD and UC patient biopsies.

## Abbreviations

IBD: Inflammatory bowel disease
CD: Crohn’s disease
UC: Ulcerative colitis
miRNA: microRNA
GWAS: Genome wide association studies
IL-10: Interleukin 10
qRT-PCR: Quantitative real-time PCR
EU: Endoscopically uninvolved
EI: Endoscopically involved
NL: Normal
