# Supplementary material for: MicroRNA signatures differentiate Crohn’s disease from ulcerative colitis
Source: BMC Immunol. 2015 Feb 10;16:5. doi: 10.1186/s12865-015-0069-0 (PMC4335694; doi:10.1186/s12865-015-0069-0)
Supplement: Additional file 1: Table S1. — MicroRNAs with statistically significant altered expression in Crohn’s disease by tissue site from pooled samples. Table S2. MicroRNAs with statistically significant altered expression in ulcerative colitis by tissue site from pooled samples. Table S3. Potential Targets for miRNAs with Elevated Expression in IBD. [file 12865_2015_69_MOESM1_ESM.docx]

| **Table S1. MicroRNAs with statistically significant altered expression in Crohn’s disease by tissue site from pooled samples** | | | | | | | | | | | | |
| --- | --- | --- | --- | --- | --- | --- | --- | --- | --- | --- | --- | --- |
|  | | **Elevated miRNAs** | | | | | **Reduced miRNAs** | | | | | |
| **miR-** | | **31** | **101** | **146a** | **375** | **N** | **21** | **31** | **146a** | **155** | **375** | **N** |
| **Tissue** | **Blood** | - | X | - | X | 2 | X | X | X | X | - | 4 |
|  | **Colon** | X | X | X | - | 3 | - | - | - | - | X | 1 |
|  | **Saliva** | - | X | - | - | 1 | - | - | - | - | - | 0 |
|  | N | 1 | 3 | 1 | 1 |  | 1 | 1 | 1 | 1 | 1 |  |

| **Table S2. MicroRNAs with statistically significant altered expression in ulcerative colitis by tissue site from pooled samples** | | | | | | | | | | | | | | | | |
| --- | --- | --- | --- | --- | --- | --- | --- | --- | --- | --- | --- | --- | --- | --- | --- | --- |
|  | | **Elevated miRNAs** | | | | | | | | | | **Reduced miRNAs** | | | | |
| **miR-** | | **19a** | **21** | **31** | **101** | **142-3p** | **142-5p** | **223** | **375** | **494** | **N** | **21** | **31** | **142-5p** | **146a** | **N** |
| **Tissue** | **Blood** | X | - | - | X | - | X | X | X | X | 6 | X | X | - | X | 3 |
|  | **Colon** | X | X | X | X | - | - | - | - | - | 4 | - | - | - | - | 0 |
|  | **Saliva** | - | X | X | - | X | - | - | - | - | 3 | - | - | X | - | 1 |
|  | N | 2 | 2 | 2 | 2 | 1 | 1 | 1 | 1 | 1 |  | 1 | 1 | 1 | 1 |  |

| TABLE S3. Potential Targets for miRNAs with Elevated Expression in IBD | | |  |
| --- | --- | --- | --- |
|  | Target | Disease-Related Function | |
| miR-21 | TLR4  KLF3/5/6/12, SMAD7  STAT3  SOCS6, TIMP3 | Innate pattern recognition receptors Transcription factors  Th17 differentiation  Signaling | |
| miR-31 | TLR4  DGCR8, DICER1  IKZF1, KLF3/13, RUNX3,  FGF7, IL34  RC3H1, TNFSF15 | Innate pattern recognition receptors  MicroRNA processing  Transcription factors  Signaling  Orchestration of immune response | |
| miR-101 | TLR4  DGCR2  DLG5  IKZF2/4, KLF3/6/8/12, RUNX1, SMAD2, STAT6  FBXW7, ING3  CCR6, JAK2  ICK, RXRB, SOCS2/5/7, TIMP3  RC3H1, TNFSF15 | Innate pattern recognition receptors  Cell migration  Barrier integrity  Transcription factors  Apoptosis/protein turnover  Th17 differentiation  Signaling  Orchestration of immune response | |
| miR-142-3p | APC  ATG16L1  IKZF1  IL6ST, RARG, SOCS6, TGFB2  NKX2-3 | Tumor suppressor  Autophagy  Transcription factors  Signaling  Orchestration of immune response | |
| miR-142-5p | SIRT7  IKZF2, IRF9, KLF10/11, SMAD2/3/4/5, SP2  ICK, IL6ST, SMURF1  RC3H1 | Chromatin remodeling  Transcription factors  Signaling  Orchestration of immune response | |
| miR-146a | CARD10  IRF5, KLF7, SMAD4, RUNX3  TLR4  RC3H1, TNFSF15 | Apoptosis/protein turnover  Transcription factors  Innate pattern recognition receptors Orchestration of immune response | |
| miR-155 | BCL10  IL23R, JAK2  RCOR1, SMAD1/2, SOCS1/6, SP1/3  CD274, FGF7, IL6ST, IL13  RC3H1 | Apoptosis/protein turnover  Th17 differentiation  Transcription factors  Signaling  Orchestration of immune response | |
| miR-223 | APC  ATG13  HDAC4  SP1/3  IL6ST  PTBP2  IL12B, PTPN2, RC3H1 | Tumor suppressor  Autophagy  Chromatin remodeling  Transcription factors  Signaling  Splicing  Orchestration of immune response | |
| miR-375 | JAK2  GATA6, IKZF4, KLF4/5/12, SP1  ING5, PIAS1  NKX2-3, RC3H1 | Th17 differentiation  Transcription factors  Signaling  Orchestration of immune response | |
| miR-494 | CARD9, ING3  DLG5  IL23R, STAT3  KLF9/11, SMAD9  SIRT1/3  SOCS6  IL12B  TLR4 | Apoptosis/protein turnover  Barrier integrity  Th17 differentiation  Transcription factors  Chromatin remodeling  Signaling  Orchestration of immune response  Innate pattern recognition receptors | |
