# Supplementary figures and images for: MicroRNA signatures differentiate Crohn’s disease from ulcerative colitis
Source: BMC Immunol. 2015 Feb 10;16:5. doi: 10.1186/s12865-015-0069-0 (PMC4335694; doi:10.1186/s12865-015-0069-0)

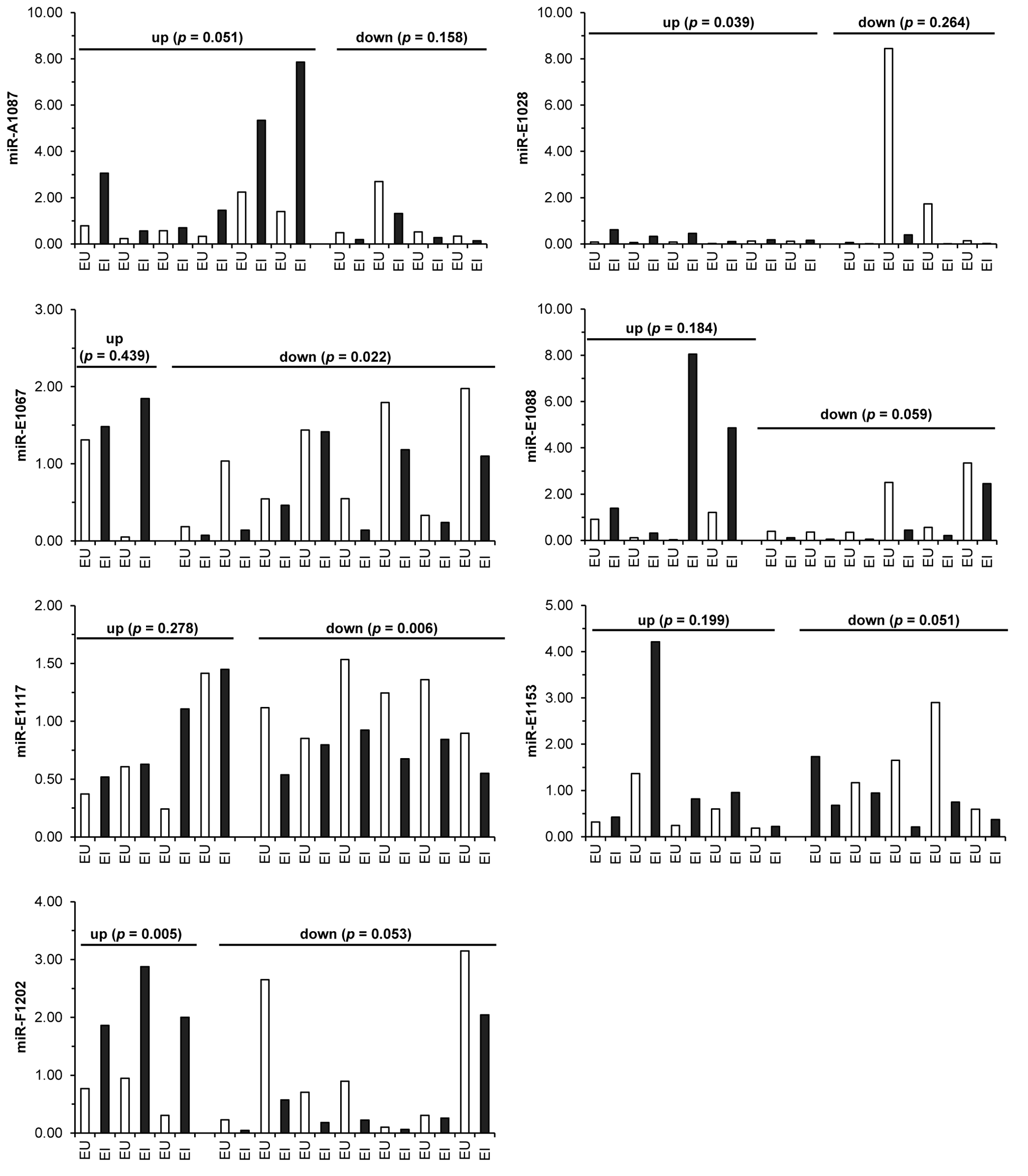

Supplement: Additional file 2: Figure S1. — miRPlus miRNA expression is altered in matched colon biopsies from Crohn’s disease subjects. Total RNA was isolated from matched endoscopically uninvolved (EU) and endoscopically involved (EI) colon biopsies from CD subjects. The RNA samples were used for SYBR Green qRT-PCR analysis for the indicated miRNAs. miRNA expression was normalized to U6 expression. Pairings were subdivided according to miRNA expression trends. Statistical significance was calculated using the Student’s paired t-test relative to the endoscopically uninvolved CD tissue. [file 12865_2015_69_MOESM2_ESM.tiff]

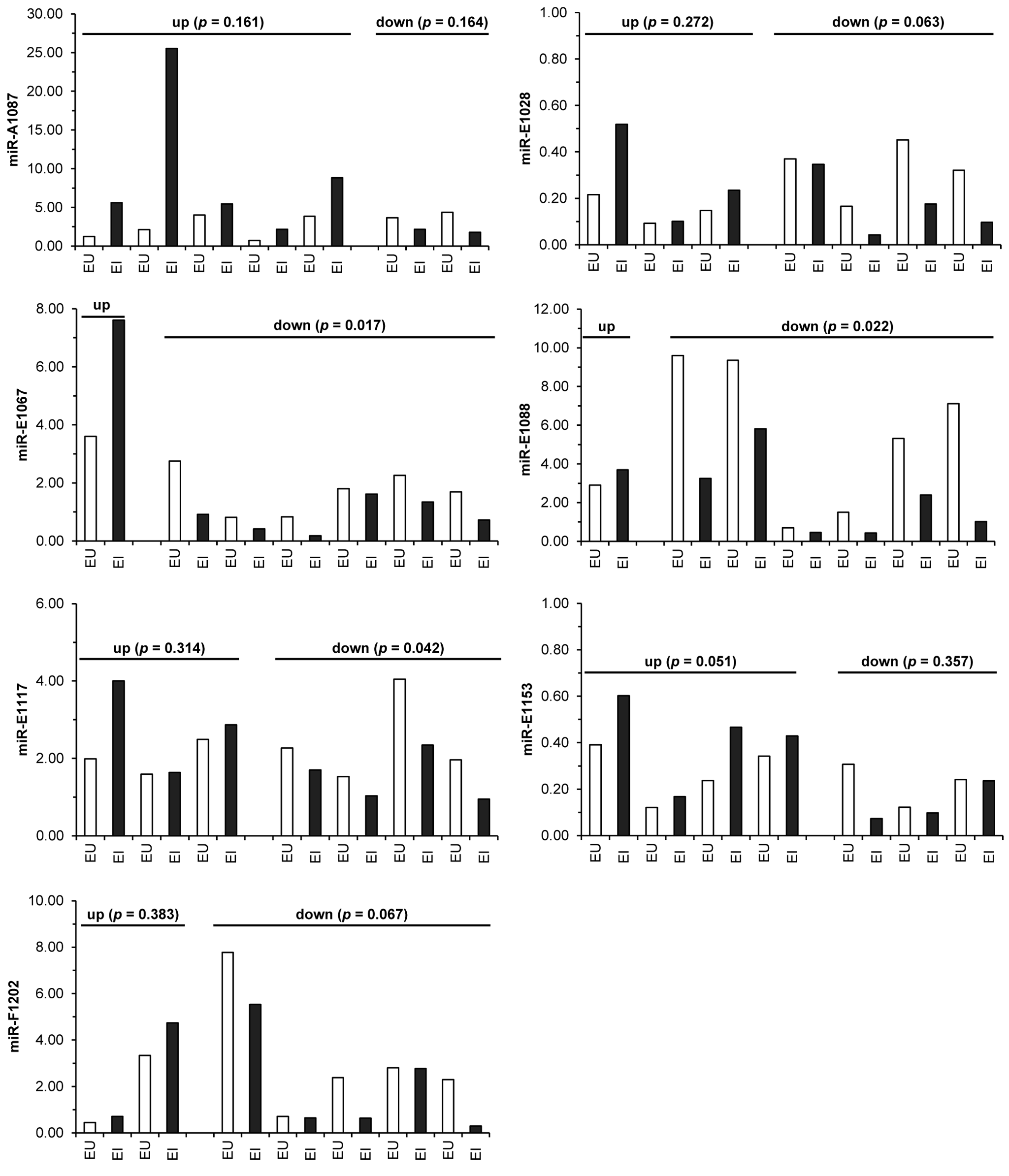

Supplement: Additional file 3: Figure S2. — miRPlus miRNA expression is altered in matched colon biopsies from ulcerative colitis subjects. Total RNA was isolated from matched endoscopically uninvolved (EU) and endoscopically involved (EI) colon biopsies from UC subjects. The RNA samples were used for SYBR Green qRT-PCR analysis for the indicated miRNAs. miRNA expression was normalized to U6 expression. Pairings were subdivided according to miRNA expression trends. Statistical significance was calculated using the Student’s paired t-test relative to the endoscopically uninvolved UC tissue. [file 12865_2015_69_MOESM3_ESM.tiff]

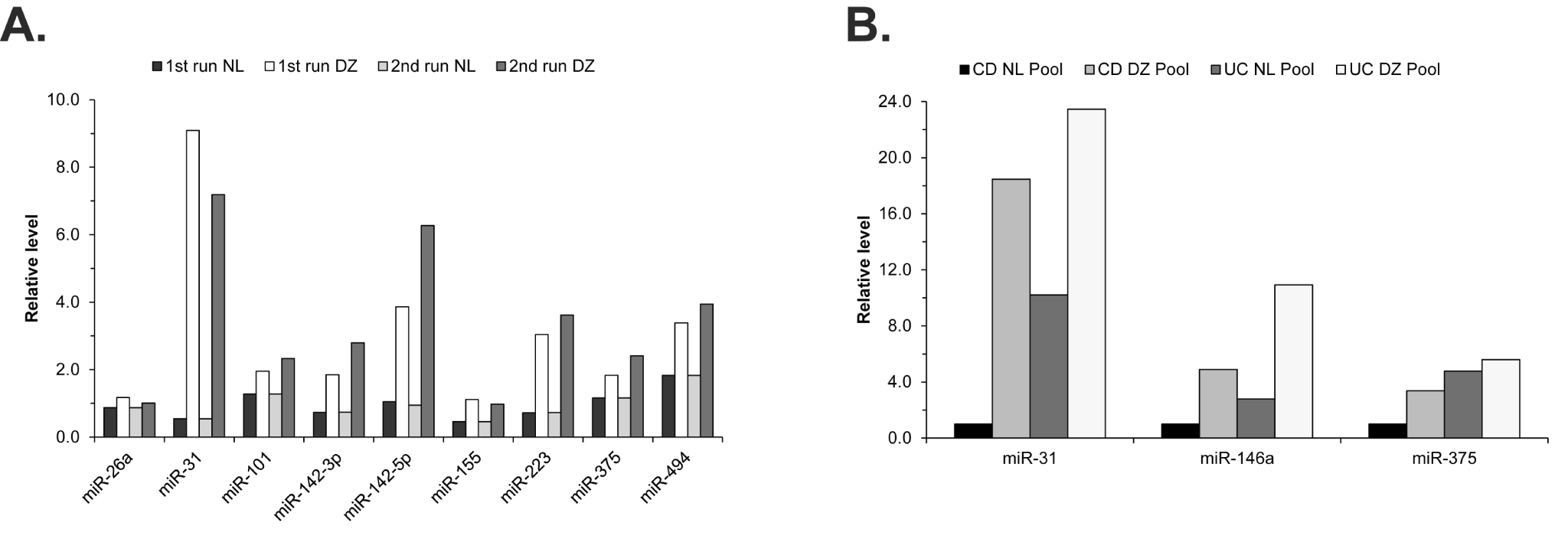

Supplement: Additional file 4: Figure S3. — Specificity and reproducibility of miRNA expression colon biopsies. (A) Replicate runs of RNA samples from matched endoscopically uninvolved versus endoscopically involved CD patient biopsies. Samples were analyzed via TaqMan qRT-PCR for the indicated miRNAs. (B) qRT-PCR analysis of miR-31, miR-146a, and miR-375 in pooled endoscopically uninvolved versus endoscopically involved CD and UC patient biopsies. [file 12865_2015_69_MOESM4_ESM.tiff]
